# When getting there is not enough: a nationwide cross‐sectional study of 998 maternal deaths and 1451 near‐misses in public tertiary hospitals in a low‐income country

**DOI:** 10.1111/1471-0528.13450

**Published:** 2015-05-14

**Authors:** OT Oladapo, OO Adetoro, BA Ekele, C Chama, SJ Etuk, AP Aboyeji, HE Onah, AM Abasiattai, AN Adamu, O Adegbola, AS Adeniran, CO Aimakhu, O Akinsanya, LD Aliyu, AB Ande, A Ashimi, M Bwala, A Fabamwo, AD Geidam, JI Ikechebelu, JO Imaralu, O Kuti, D Nwachukwu, L Omo‐Aghoja, K Tunau, J Tukur, OUJ Umeora, AC Umezulike, OA Dada, Ӧ Tunçalp, JP Vogel, AM Gülmezoglu, Nigeria Near‐miss and Maternal Death Surveillance Network, OO Fakeye, D Abubakar, A Adeniyi, N Adewole, A Adeyemi, A Agbata, B Ageda, ES Aigere, FC Anolue, A Bariweni, F Ezugwu, B Fawole, K Hunyinbo, C Kamanu, G Maduakor, A Massa, JY Moruppa, EA Nzeribe, AN Ocheke, AO Oguntayo, T Onile, JO Sotunsa, GO Ugwu, I Wanonyi

**Affiliations:** ^1^ Department of Reproductive Health and Research including UNDP/UNFPA/UNICEF/WHO/World Bank Special Programme of ResearchDevelopment and Research Training in Human Reproduction (HRP)World Health OrganizationGenevaSwitzerland; ^2^ Department of Obstetrics and GynaecologyOlabisi Onabanjo University Teaching HospitalSagamuNigeria; ^3^ Department of Obstetrics and GynaecologyUniversity of Abuja Teaching HospitalGwagwaladaNigeria; ^4^ Department of Obstetrics and GynaecologyUniversity of Maiduguri Teaching HospitalMaiduguriNigeria; ^5^ Department of Obstetrics and GynaecologyUniversity of Calabar Teaching HospitalCalabarNigeria; ^6^ Department of Obstetrics and GynaecologyUniversity of Ilorin Teaching HospitalIlorinNigeria; ^7^ Department of Obstetrics and GynaecologyUniversity of Nigeria Teaching HospitalEnuguNigeria; ^8^ Department of Obstetrics and GynaecologyUniversity of Uyo Teaching HospitalUyoNigeria; ^9^ Department of Obstetrics and GynaecologyFederal Medical CentreBirnin‐KebbiNigeria; ^10^ Department of Obstetrics and GynaecologyLagos University Teaching HospitalIdi‐ArabaNigeria; ^11^ Department of Obstetrics and GynaecologyUniversity College HospitalIbadanNigeria; ^12^ Department of Obstetrics and GynaecologyFederal Medical CentreOwoNigeria; ^13^ Department of Obstetrics and GynaecologyAbubakar Tafawa Balewa University Teaching HospitalBauchiNigeria; ^14^ Department of Obstetrics and GynaecologyUniversity of Benin Teaching HospitalBenin‐CityNigeria; ^15^ Department of Obstetrics and GynaecologyFederal Medical CentreBirnin‐KuduNigeria; ^16^ Department of Obstetrics and GynaecologyFederal Medical CentreNguruNigeria; ^17^ Department of Obstetrics and GynaecologyLagos State University Teaching HospitalIkejaNigeria; ^18^ Department of Obstetrics and GynaecologyNnamdi Azikwe University Teaching HospitalNnewiNigeria; ^19^ Department of Obstetrics and GynaecologyObafemi Awolowo University Teaching Hospital ComplexIle‐IfeNigeria; ^20^ Department of Obstetrics and GynaecologyFederal Medical CentreBidaNigeria; ^21^ Department of Obstetrics and GynaecologyDelta State University Teaching HospitalAbrakaNigeria; ^22^ Department of Obstetrics and GynaecologyUsmanu DanFodiyo University Teaching HospitalSokotoNigeria; ^23^ Department of Obstetrics and GynaecologyAminu Kano University Teaching HospitalKanoNigeria; ^24^ Department of Obstetrics and GynaecologyFederal University Teaching HospitalAbakalikiNigeria; ^25^ Department of Obstetrics and GynaecologyNational HospitalAbujaNigeria; ^26^ Centre for Research in Reproductive HealthSagamuNigeria

**Keywords:** Clinical audit, maternal death, maternal near miss, quality of care, severe acute maternal morbidity, WHO near‐miss criteria

## Abstract

**Objective:**

To investigate the burden and causes of life‐threatening maternal complications and the quality of emergency obstetric care in Nigerian public tertiary hospitals.

**Design:**

Nationwide cross‐sectional study.

**Setting:**

Forty‐two tertiary hospitals.

**Population:**

Women admitted for pregnancy, childbirth and puerperal complications.

**Methods:**

All cases of severe maternal outcome (SMO: maternal near‐miss or maternal death) were prospectively identified using the WHO criteria over a 1‐year period.

**Main outcome measures:**

Incidence and causes of SMO, health service events, case fatality rate, and mortality index (% of maternal death/SMO).

**Results:**

Participating hospitals recorded 91 724 live births and 5910 stillbirths. A total of 2449 women had an SMO, including 1451 near‐misses and 998 maternal deaths (2.7, 1.6 and 1.1% of live births, respectively). The majority (91.8%) of SMO cases were admitted in critical condition. Leading causes of SMO were pre‐eclampsia/eclampsia (23.4%) and postpartum haemorrhage (14.4%). The overall mortality index for life‐threatening conditions was 40.8%. For all SMOs, the median time between diagnosis and critical intervention was 60 minutes (IQR: 21–215 minutes) but in 21.9% of cases, it was over 4 hours. Late presentation (35.3%), lack of health insurance (17.5%) and non‐availability of blood/blood products (12.7%) were the most frequent problems associated with deficiencies in care.

**Conclusions:**

Improving the chances of maternal survival would not only require timely application of life‐saving interventions but also their safe, efficient and equitable use. Maternal mortality reduction strategies in Nigeria should address the deficiencies identified in tertiary hospital care and prioritise the prevention of severe complications at lower levels of care.

**Tweetable abstract:**

Of 998 maternal deaths and 1451 near‐misses reported in a network of 42 Nigerian tertiary hospitals in 1 year.

## Tweetable abstract

Of 998 maternal deaths and 1451 near‐misses reported in a network of 42 Nigerian tertiary hospitals in 1 year.

## Introduction

Despite the substantial progress made towards reducing global maternal mortality over the last decade, it is apparent that the fifth Millennium Development Goal (MDG‐5) will not be reached. More than a quarter of a million women lost their lives to preventable pregnancy and childbirth complications in 2013.^1^ The global strategy to reduce pregnancy‐related deaths has mainly focused on increasing skilled birth attendance with the aim of expanding coverage of effective interventions during childbirth. Yet, the increasing proportion of institutional births in recent years has not been matched by quality health services.^2^ Available data on maternal deaths occurring in hospitals in low‐income countries suggest that a focus on quality of care should complement universal promotion of skilled birth attendance in the post‐MDG era.^3^

One major reason why some countries are making little or no progress towards achieving MDG‐5 is the lack of reliable data on which to measure progress and initiate action. Maternal death and, in the last decade, maternal near miss, have become standard measures of quality of care on which progress can be assessed.^4^, ^5^, ^6^, ^7^, ^8^, ^9^ Unfortunately, countries with the highest burden of maternal mortality and morbidity have the least reliable data on such health indicators. For instance, in Nigeria, there are no population‐based data on maternal death at the country level and the vital registration system is currently unable to provide reliable estimates. The country relies on estimates derived from statistical modelling by international agencies, which are often insufficient to assess the quality of care, monitor trend on the short term or determine national health system priorities.

The main goal of this study, the *Nigeria Near‐miss and Maternal Death Survey*, was to assess the burden of maternal death and near‐miss and review associated health service events at the highest level of health care delivery in Nigeria. We achieved this through prospective surveillance and data collection on life‐threatening maternal complications (severe maternal outcomes, SMO) in a nationwide network of Nigerian public tertiary institutions. Specifically, we assessed the frequencies and cause distribution of maternal deaths and near‐misses, areas of substandard care provision for women experiencing SMO, and overall performance of care using standard indicators of quality of care. We explored avoidable factors affecting maternal survival by comparing health service events surrounding the management of cases of near‐misses with those of maternal deaths.

## Methods

### Design, setting and population

The study protocol and other methodological considerations have been published in detail previously.^10^ Here, we describe the general outline of the study methods and highlight revisions in the study procedures that were informed by the WHO Human Reproduction Programme (HRP) *Research Project Review Panel* and WHO Ethics Review Committee after the publication of the protocol. In brief, our study was a nationwide multicentre cross‐sectional study that identified women who died or suffered a maternal near‐miss from pregnancy, childbirth or puerperal complications based on uniform identification criteria. All (46) public tertiary hospitals providing obstetric services (University hospitals and Federal Medical Centres) within the six geopolitical zones of Nigeria were targeted for inclusion in the study. Of these, 42 hospitals provided consent, participated and successfully implemented the study. All women admitted for delivery or within 42 days of delivery or spontaneous loss/termination of pregnancy over a period of 1 year in all participating hospitals made up the study population. Through a prospective surveillance, cases of maternal death and maternal near‐miss during the period that the women remain admitted to hospital were identified and included in the study. The local research team analysed and documented the health service events (processes) surrounding the care of every woman identified using a structured format.

We defined maternal near‐miss, SMO, and other near‐miss indicators according to the WHO (Supporting Information Appendix S1).^11^, ^12^, ^13^ Women were identified as a maternal near‐miss if they met the WHO near‐miss criteria (Supporting Information Appendix S2).^12^ Maternal death was defined according to International Classification of Diseases (ICD‐10).^13^ Individual level data were not extracted for women without SMO but we used a structured form to obtain monthly records of the total number of deliveries, live births, stillbirths, and distribution of all maternal complications managed at the facilities regardless of their severity and final outcome.

The WHO HRP research project review panel reviewed and approved the scientific content of the study (protocol ID, A65745). The WHO Research Ethics Review Committee and ethical review authorities in all participating hospitals reviewed and approved the study. Individual level written consent was not required for this study as there was no interview of women or medical staff.

### Study procedures

We collected data continuously for a period of 1 year at each hospital. Each facility identified a clinician (resident doctor) from a department other than that designated for obstetric services (e.g. paediatrics or general practice), who was trained as the data collector. The data collector was not involved in the routine provision of care for obstetric patients in the hospital but had sufficient experience in clinical obstetrics (including prior clinical rotation through the obstetric unit) to handle the data collection process. On a daily basis, the data collector visited the obstetric ward, gynaecological/abortion care unit, labour room, emergency and intensive care units and examined medical records to identify eligible women. The data collector completed a simple individual‐level data form for all eligible women (and their babies) within 24 hours of identifying an SMO. At the time of hospital discharge (or death), the data collector updated the information for the period that the women remained on admission on the same form. To maintain continuous surveillance and data collection, appropriate time was allotted to each data collector to participate in the study, and each hospital trained an additional data collector to serve as a substitute when needed. A hospital coordinator (consultant obstetrician) supervised the data collector and verified that all cases meeting the study inclusion criteria were identified.

We obtained data on demographic and reproductive characteristics, admission history, and markers of organ dysfunction underlying maternal near‐miss or death. For every woman identified, we collected information on the primary complication (i.e. underlying cause) that triggered the maternal near‐miss or death (Appendix S1), the time interval between diagnosis of this complication and ‘definitive treatment/intervention’ required to avert death, the level of the most senior medical personnel who attended to the woman, and the time until such personnel physically intervened in the management.

For the purpose of this study, we defined ‘definitive treatment/intervention’ as the most crucial intervention (or combination of interventions) required to actually end or reverse the underlying pathological process and avert death, and not important but mainly supportive interventions. For instance, while fluid management was equally important, the definitive treatment for eclampsia was taken as prevention of further fits with magnesium sulphate and delivery of the fetus; for maternal sepsis, the administration of systemic antibiotics; for postpartum haemorrhage (PPH), the use of therapeutic uterotonic or hysterectomy depending on its cause and progression; and for obstructed labour, caesarean section to relieve the obstruction. We estimated the time interval (in minutes) between diagnosis of the disease condition and initiation of definitive treatment/intervention. For example, we estimated the time between the diagnosis of eclampsia and the first dose of magnesium sulphate where eclampsia was the underlying cause of an SMO. We developed a manual of operations, which was used by the local research team to ensure consistent judgement and interpretation of study procedures across sites.

All data were handled centrally by the Data Management Unit of the Centre for Research in Reproductive Health, Sagamu, Nigeria. The study principal investigator performed visual validity crosschecks of all forms received at the central coordinating unit before data entry. Individual level data were doubly entered into a computer‐based data management system that was developed by Centro Rosarino de Estudios Perinatales (CREP, Rosario, Argentina). Data quality was ensured and maintained through in‐built validation procedures in the data management system, which allowed prompt identification and treatment of inconsistencies.

### Data analysis

We conducted descriptive analysis of the demographic and reproductive characteristics, mode and timing of hospital admission, stratified according to the type of SMO. We determined the frequencies of organ dysfunction markers among women with SMO. We calculated the total live births and stillbirths, maternal near‐miss ratio, severe maternal outcome ratio, and intrahospital maternal mortality ratio, at the facility, regional and country levels (as defined in Appendix S1).^11^, ^12^ We presented the frequencies of the primary complications underlying the occurrence of maternal near‐miss, maternal death and SMO. We assessed overall care performance for life‐threatening complications and direct obstetric complications by estimating mortality index (MI)^11^ and cause‐specific case fatality rates,^14^ respectively (as defined in Appendix S1). We expressed variables related to time intervals as median (with interquartile range, IQR) and compared findings between cases of maternal near‐miss and maternal death overall and according to key groups of complications. Categorical variables were compared with the chi‐squared test, Fisher's exact test and odds ratio (OR) as appropriate. The *t*‐test and Mann–Whitney test were used to compare normally and non‐normally distributed continuous variables, respectively. We considered differences between observations as statistically significant when the *P*‐value was <0.05. Statistical analyses were performed using EPI INFO 7.1.4 (CDC, USA) and MEDCALC for Windows, version 13.3.1 (MedCalc Software, Ostend, Belgium, www.medcalc.org). Routine obstetric data from all hospitals were analysed using Microsoft EXCEL software.

## Results

Each study facility completed a 12‐month surveillance between 1 June 2012 and 14 August 2013. A total of 100 107 women admitted for pregnancy, childbirth or puerperal complications were included in the surveillance. Most of these hospitals were located in urban and peri‐urban areas across Nigeria with very similar facility characteristics in terms of staffing, resources and availability of services (data not shown). Among women presenting to these facilities, a total of 97 634 births were recorded: 91 724 live births and 5910 stillbirths. Thus, the stillbirth rate for all participating hospitals was 60.5 per 1000 births.

During the surveillance period, 998 women who died and 1451 women who survived organ dysfunction (maternal near‐miss) while under hospital care were included in the database, giving a total of 2449 cases of SMO. Apart from those who died while in the hospital, 136 women who were already dead as a result of pregnancy‐related complications before arriving at the hospital were documented but not included in the SMO database. Intra‐hospital maternal mortality ratio (MMR) was 1088 per 100 000 live births (1.1%), maternal near‐miss ratio was 15.8/1000 live births (1.6%) and severe maternal outcome ratio was 26.7/1000 live births (2.7%).

Supporting Information Table S1 shows that the demographic characteristics and past reproductive history were generally comparable between women who died and those who survived organ dysfunction (i.e. *P *> 0.05) except for place of residence >5 km, which was more frequent among women who died (70.9 versus 64.1%; *P* < 0.05). Three‐quarters of the women were not registered for antenatal care and delivery at the participating hospital. Over half of the women came as referrals from surrounding health facilities. A total of 2333 (91.8%) were admitted in critical condition, with close to half of them arriving at night.

**Figure 1:**
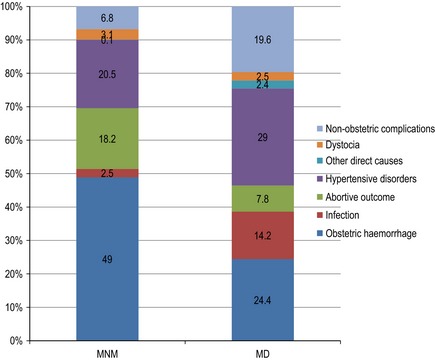
Relative contribution of key groups of complications by outcome severity.

Table 1 shows the distribution of the primary complication resulting in organ dysfunction associated with maternal near‐miss or death and the corresponding MI. Obstetric haemorrhage (39.0%) and hypertensive disorders (24.0%) were the most frequent groups of complications resulting in SMO. In terms of specific disease conditions, eclampsia was the most frequent complication, followed by PPH, ruptured uterus, placenta abruption, and ruptured ectopic gestation in descending order. Eclampsia accounted for one‐fifth of all maternal deaths, twice as many deaths that resulted from PPH. Figure 1 shows the relative contribution of key groups of complications according to the severity of maternal outcomes. Hypertensive disorders (29.0%), obstetric haemorrhage (24.4%) and indirect causes (19.6%) were the greatest contributors to maternal death. The contributions of infections and indirect causes to maternal death were disproportionally larger than their contributions to maternal near‐miss (14.2 versus 2.5% and 19.6 versus 6.8%, respectively). Table 1 shows that the overall MI for women with any life‐threatening condition was 40.8%. Mortality indices were generally high across the key groups of complications but were worst for infections and indirect causes of SMO.

**Table 1:** Distribution of primary complications^a^ resulting in organ dysfunction associated with maternal near‐miss or maternal death

Primary complication	Maternal near‐miss (MNM) *n *=* *1451	Maternal death (MD) *n *=* *998	Severe maternal outcome (SMO) *n *=* *2449	Mortality index (% of MD/SMO) 40.8
**Obstetric haemorrhage**	**711 (49.0)**	**244 (24.4)**	**956 (39.0)**	**25.5**
Placenta praevia	83 (5.7)	12 (1.2)	95 (3.9)	12.6
Placental abruption	174 (12.0)	36 (3.6)	210 (8.6)	17.1
Morbidly adherent placenta	19 (1.3)	2 (0.2)	21 (0.9)	9.5
Ruptured uterus	175 (12.1)	87 (8.7)	262 (10.7)	33.2
Postpartum haemorrhage	251 (17.3)	103 (10.3)	354 (14.5)	29.1
Other obstetric haemorrhage	9 (0.6)	4 (0.4)	13 (0.5)	30.8
**Pregnancy‐related infection**	**37 (2.5)**	**142 (14.2)**	**179 (7.3)**	**79.3**
Puerperal genital sepsis	24 (1.7)	67 (6.7)	91 (3.7)	73.6
Chorioamnionitis	5 (0.3)	17 (1.7)	22 (0.9)	77.3
Pyelonephritis	2 (0.1)	2 (0.2)	4 (0.2)	50.0
Sepsis/systemic infections	6 (0.4)	56 (5.6)	62 (2.5)	90.3
**Abortive outcome**	**264 (18.2)**	**78 (7.8)**	**342 (14.0)**	**22.8**
Abortion‐related haemorrhage	55 (3.8)	19 (1.9)	74 (3.0)	25.7
Abortion‐related infection	35 (2.4)	46 (4.6)	81 (3.3)	56.8
Ruptured ectopic	174 (12.0)	13 (1.3)	187 (7.6)	7.0
**Hypertensive disorders**	**298 (20.5)**	**289 (29.0)**	**587 (24.0)**	**49.2**
Chronic hypertension	8 (0.6)	7 (0.7)	15 (0.6)	46.7
Pre‐eclampsia	71 (4.9)	69 (6.9)	140 (5.7)	49.3
Eclampsia	219 (15.1)	213 (21.3)	432 (17.6)	49.3
**Dystocia**	**45 (3.1)**	**25 (2.5)**	**70 (2.9)**	**35.7**
Prolonged labour	14 (1.0)	6 (0.6)	20 (0.8)	30.0
Labour obstruction	31 (2.1)	19 (1.9)	50 (2.0)	38.0
**Other direct obstetric complications**	**1 (0.1)**	**24 (2.4)**	**25 (1.0)**	**96.0**
Embolism^b^	1 (0.1)	24 (2.4)	25 (1.0)	96.0
**Non‐obstetric (indirect) complications**	**95 (6.5)**	**196 (19.6)**	**291 (11.9)**	**67.4**
HIV/AIDS/HIV wasting syndrome	7 (0.5)	41 (4.1)	48 (2.0)	85.4
Malaria	9 (0.6)	12 (1.2)	21 (0.9)	57.1
Anaemia	47 (3.2)	63 (6.3)	110 (4.5)	57.3
Heart disease	17 (1.2)	20 (2.0)	37 (1.5)	54.1
Lung disease	4 (0.3)	8 (0.8)	12 (0.5)	66.7
Renal disease	4 (0.3)	4 (0.4)	8 (0.3)	50.0
Hepatic disease	2 (0.1)	9 (0.9)	11 (0.4)	81.8
Cancer	0 (0.0)	11 (1.1)	11 (0.4)	100.0
Other conditions/complications	5 (0.3)	28 (2.8)	33 (1.3)	84.8

a All identified causes are mutually exclusive.

b Thromboembolism, amniotic fluid embolism and air embolism.

Supporting Information Table S2 shows the regional distribution of direct obstetric complications and the corresponding cause‐specific case fatality rates (CFR). Cause‐specific CFR was >1% for the majority of direct obstetric complications and ranged between 0.5 and 15.3%. Cause‐specific CFR was very high (>10%) for ruptured uterus, eclampsia, puerperal sepsis and systemic infections. There was a good correlation between mortality indices and cause‐specific CFR for direct obstetric complications (Pearson *r* = 0.7). When these complications were further explored to identify the underlying organ dysfunctions, cardiovascular, respiratory and coagulation dysfunctions were the most frequent organ dysfunctions occurring in women with SMO (table not shown). In general, the mortality indices were poor for all categories of organ dysfunctions but were worse for renal and respiratory dysfunctions (64.3 and 63.3%, respectively). Multiple organ dysfunctions was over twice as frequent among cases of maternal death as compared with maternal near‐misses (93.4 versus 43.9%).

Supporting Information Table S3 summarises the maternal and perinatal outcomes and near‐miss indicators by regions. The severe maternal outcome ratio ranged between 21.1/1000 live births in the northcentral region and 36.5/1000 live births in the southwest region of Nigeria. Forty‐seven percent of all stillbirths were recorded in the northeast and northwest regions. Mortality indices were highest for health facilities in the northwest and southwest regions (49.4 and 42.5%, respectively). The southeast and southwest regions recorded the largest number of maternal deaths before arrival at the hospital. Supporting Information Table S4 shows the intrahospital MMR (with their 95% confidence intervals) by participating hospitals. All hospitals recorded maternal death and there was a considerable overlap in the confidence intervals of intrahospital MMR across the majority of these hospitals (Supporting Information Figure S1).

As shown in Table 2, there was a variable length of time between the diagnosis of underlying cause of SMO and ‘definitive treatment/intervention’. A total of 2325 (94.9%) women with SMO were documented to have received ‘definitive treatment/intervention’: 1401 women (96.6%) with maternal near‐miss and 924 women (92.5%) who died. Although the median time interval to initiate this intervention for all cases of SMO was 60 minutes (IQR: 21–215 minutes), it was over 4 hours in more than one‐fifth of cases. In 47.4% of all cases of SMO, the interval was more than 1 hour. Overall, median time interval was 17 minutes shorter among those who died compared with those who survived. The proportion of women who died was statistically significantly higher than that of the survivors among women who received definitive interventions within half an hour (44.1 versus 33.3%, *P* < 0.0001) and after 4 hours (25.5 versus 19.3%, *P* = 0.0004) of diagnosis of underlying cause of organ dysfunction. When the median time was compared according to the groups of disease condition underlying SMO, no differences were observed between cases of maternal near‐misses and maternal death. In 82.9% of cases, the most senior cadre of medical staff who was physically present to attend to the woman at the time of near‐miss experience or events leading to death was a senior registrar or consultant (table not shown). Overall, the median time interval between diagnosis of underlying cause of SMO and attention by this cadre of staff was 60 minutes (IQR: 21–219 minutes) but was longer than 4 hours in close to a quarter of cases. This median time interval between diagnosis and attention by the senior personnel was not significantly different between cases of maternal near‐miss and maternal death (*P *= 0.8767). However, among the sample of SMOs seen after 4 hours of diagnosis, cases of maternal death were significantly more frequent compared with near‐misses (26.2 versus 20.9%, *P *= 0.0031).

**Table 2:** Time between diagnosis of the primary cause of severe maternal outcomes and intervention to avert maternal death and attention by senior personnel by severity of outcomes

Time to definitive intervention (minutes)	Severe maternal outcome (SMO) *n = *2325 (%)	Maternal near‐miss (MNM) *n = *1401 (%)	Maternal death (MD) *n = *924 (%)	*P*‐value (MNM versus MD)
≤30	874 (37.5)	467 (33.3)	407 (44.1)	<0.0001
31–60	351 (15.1)	233 (16.6)	117 (12.7)	0.0104
61–120	330 (14.2)	240 (17.1)	90 (9.7)	<0.0001
121–180	145 (6.2)	104 (7.4)	41 (4.4)	0.0047
181–240	119 (5.1)	86 (6.1)	33 (3.6)	0.0079
>240	509 (21.9)	271 (19.3)	236 (25.5)	0.0004
All SMO, median (IQR)	60 (21 ‐215)	62 (30‐186)	45 (20‐250)	0.0319

Time to intervention by primary cause of organ dysfunction (minutes)	*n*	*n*	*n*	
	Median (IQR)	Median (IQR)	Median (IQR)	
Obstetric haemorrhage	919	688	231	
	60 (22–182.5)	60 (25–180)	50 (20–180)	0.1714
Pregnancy‐related infection	169	34	135	
	45 (20–242.5)	77.5 (20–235)	45 (22.5–315)	0.9734
Abortive outcome	331	257	74	
	90 (33–243)	110 (40–210)	60 (20–259.5)	0.2228
Hypertensive disorders	561	287	273	
	45 (15–242.5)	60 (17.5–202.5)	30 (12.5–252)	0.2902
Dystocia	69	45	24	
	120 (57.5–361)	120 (52.5–210)	250.5 (65–402)	0.0998
Other direct obstetric causes	24	1	23	
	15 (5–40)	5 (5–5)	15 (7.5–52.5)	0.3815
Non‐obstetric (indirect) complications	253	88	165	
	80 (25–370)	120 (30–325)	75 (23–430)	0.6537

Time to attention by senior personnel (minutes)	*n = *2325 (%)	*n = *1386 (%)	*n = *939 (%)	
≤30	949 (40.8)	556 (40.1)	393 (41.9)	0.4275
31–60	404 (17.4)	264 (19.1)	140 (14.9)	0.0114
61–120	245 (10.5)	157 (11.3)	88 (9.4)	0.1503
121–180	122 (5.2)	75 (5.4)	47 (5.0)	0.7369
181–240	70 (3.1)	45 (3.3)	25 (2.7)	0.6115
>240	535 (23.0)	289 (20.9)	246 (26.2)	0.0031
All SMO, median (IQR)	60 (20–219)	60.0 (21–183)	60.0 (21–320)	0.8767

The local research team identified deficiencies in the clinical management of close to half of SMO cases (*n = *1215; 49.6%). ‘Any deficiency in care provision’ was more common among women who died compared with women with near‐misses (MD: 596/998, 59.8% versus MNM: 619/1451, 42.6%; *P *< 0.0001. Table 3 shows the frequencies of avoidable factors associated with substandard care according to severity of outcomes. Late presentation to the hospital (35.3%), lack of health insurance/inability to pay for required services (17.5%), and non‐availability of required blood/blood products (12.7%) were identified as leading contributors to substandard care for women with SMO. Specific problems identified were generally more frequent among women who died than those who survived, but when considered as categories, ‘any patient‐orientated problems’ and ‘medical personnel problems’ were statistically different between the two groups.

**Table 3:** Identified problems^a^ associated with deficiencies in care for women with life‐threatening conditions

Problem	SMO *n *=* *2449	MNM *n* = 1451	MD *n *=* *998	Unadjusted OR (95% CI)	*P*‐value (MNM versus MD)
**Administrative problems**					
Power supply	107 (4.4)	74 (5.1)	33 (3.3)	1.57 (1.03–2.38)	0.0331
Transport and or communication	78 (3.2)	50 (3.4)	28 (2.8)	1.24 (0.77–1.98)	0.3753
Life‐saving drugs in hospital pharmacy	68 (2.8)	35 (2.4)	33 (3.3)	0.72 (0.44–1.17)	0.1856
Blood/blood products	312 (12.7)	204 (14.0)	108 (10.8)	1.34 (1.05–1.73)	0.0182
Equipment/competent staff	124 (5.1)	50 (3.4)	74 (7.4)	0.29 (0.20–0.41)	<0.0001
Other administrative	121 (4.9)	57 (3.9)	64 (6.4)	0.60 (0.41–0.86)	0.0053
Any administrative problem	478 (19.5)	279 (19.2)	199 (19.9)	0.95 (0.78–1.17)	0.6623
**Patient‐orientated problems**					
Late presentation to hospital	866 (35.3)	413 (28.4)	453 (45.3)	0.48 (0.40–0.57)	<0.0001
Refuse treatment	119 (4.9)	70 (4.8)	49 (4.9)	0.98 (0.67–1.43)	0.9229
Language barrier	26 (1.1)	15 (1.0)	11 (1.1)	0.94 (0.43–2.04)	0.8703
Lack of insurance/inability to pay for intervention	429 (17.5)	228 (15.7)	201 (20.1)	0.73 (0.60–0.91)	0.0037
Other patient‐orientated problems	113 (4.6)	58 (4.0)	55 (5.5)	0.71 (0.48–1.04)	0.0790
Any patient‐orientated problem	1032 (42.1)	523 (36.0)	509 (51.0)	0.53 (0.46–0.64)	<0.0001
**Medical personnel problems**					
Delay in diagnosis	166 (6.8)	67 (4.6)	99 (9.9)	0.43 (0.31–0.60)	<0.0001
Delay in treatment	217 (8.9)	85 (5.8)	132 (13.2)	0.40 (0.30–0.53)	<0.0001
No assessment by senior doctor	88 (3.6)	21 (1.4)	67 (6.7)	0.20 (0.12–0.36)	<0.0001
Poor monitoring	116 (4.7)	29 (2.0)	87 (8.7)	0.21 (0.14–0.33)	<0.0001
Other medical problems	53 (2.2)	20 (1.4)	33 (3.3)	0.41 (0.23–0.72)	0.0021
Any medical personnel problem	422 (17.2)	165 (11.4)	257 (25.8)	0.39 (0.31–0.48)	<0.0001

MD, maternal death; MNM, maternal near‐miss; OR: odds ratio; SMO, severe maternal outcome.

a Identified problems are not mutually exclusive.

## Discussion

### Main findings

Our study shows that an unacceptably high number of maternal deaths occur in Nigerian public tertiary hospitals annually. The overall performance of these hospitals as indicated by the survival rate of women with life‐threatening complications was suboptimal, although some important regional differences exist. The majority of women presenting with life‐threatening conditions received essential interventions to avert maternal death but with substantial delays in many cases. Although obstetric haemorrhage and hypertensive disorders were the most frequent causes of organ dysfunctions, the survival rates following infections and indirect complications were considerably lower.

### Strengths and limitations

To our knowledge, our investigation reports the largest cohort of maternal deaths prospectively identified in any hospital‐based study and the first attempt to date to measure precisely third phase delay contributing to adverse maternal outcomes.^15^, ^16^ The study was the largest simultaneous investigation of maternal near‐miss and death based on standardised and uniform definitions in any African country. According to the latest maternal mortality estimate for Nigeria,^1^ we were able to capture events surrounding about 2.5% of all maternal deaths that occurred in Nigeria between 2012 and 2013, when the study was conducted. Nonetheless, the absolute number of recorded deaths clearly indicates the public health impact of maternal mortality in Nigeria.

A number of limitations need to be highlighted. Despite our efforts to ensure accurate implementation of study procedures and high quality data, it was possible that the large number of health institutions, staff, laboratory capacities, medical protocols and records formats and new terminologies may have resulted in misclassification and affected the timing of some obstetric events and interventions. Another limitation was that this study was performed in public‐funded tertiary facilities and therefore the data might not reflect the standard of care in lower level and private hospitals.

### Interpretation

The burden of maternal near‐miss and SMO in this cohort is much higher than the findings from other large surveillance networks.^8^, ^17^, ^18^ In our study, maternal near‐miss and SMO accounted for 1.6 and 2.7% respectively, compared with 0.1 and 0.2% reported by the Argentinian network,^8^ 0.9 and 1.1% by the Brazilian network,^17^ and 0.8 and 1.0% by the WHO multicountry survey (WHO MCS) network.^18^ The incidence of maternal near‐miss was much higher than that reported in smaller studies from Africa and other world regions.^19^ Given the large proportion of women arriving in a critical state in our study, and those who died before arrival, this excess burden suggests significant deficiencies in the prevention, identification and referral of severe morbidities at lower level and private hospitals, as well as barriers to appropriate care‐seeking at the community level.^20^

We found an overall mortality index of 41%, suggesting that on average, about four of every ten women developing organ dysfunction did not survive the underlying complication. This index is about 2.5 times higher than that reported in the Brazilian^17^ and WHO MCS^18^ networks and suggests that the quality of care provided for women at these tertiary hospitals needs further improvement. One reason for the suboptimal care observed in our study might be the case mix of women presenting to the tertiary facilities in Nigeria, with a higher prevalence of SMO, compared with those in the Brazilian and WHO MCS networks. It would be fair to infer that a minimal increase in the incidence of women presenting with life‐threatening conditions is likely to result in excess maternal mortality in hospitals with moderate capacity to provide comprehensive care (i.e. the more the cases of organ dysfunction, the fewer women saved).

Similar to observations in other studies,^17^, ^18^, ^21^ obstetric haemorrhage and hypertensive disorders were responsible for over half of maternal near‐misses and deaths, confirming their persistent role as lead contributors to preventable maternal death also at the facility level. However, the performance of the hospitals in our study in terms of women surviving specific complications was better for obstetric haemorrhage than hypertensive disorders. Approximately half of all women with organ dysfunction due to any of the pregnancy hypertensive syndromes died, suggesting an even wider gap in the care provided for these women. Any effort to significantly reduce maternal death must therefore include prevention and prompt management of organ dysfunction resulting from hypertensive disorders. Although the hospitals fared better with respect to managing haemorrhagic complications, further reduction in maternal death can still be achieved by addressing the deficiencies in blood transfusion services, as observed in high‐income countries.^22^

WHO MCS showed that essential interventions to manage severe morbidities are available and used in health facilities even in high MMR countries such as Nigeria.^18^ In the current study, we estimated the time that critical interventions were initiated to assess whether it made any difference to maternal survival. Although we anticipated variable distribution of the time intervals between diagnosis of disease conditions and appropriate interventions, the long delays before such interventions were provided is a cause for concern. However, the comparison of the overall response time between near‐miss and maternal death (which indicates shorter response time for maternal deaths than for near‐misses) needs to be interpreted with caution. One explanation for this unusual finding may be related to our definition of ‘definitive intervention’, which for pragmatic reasons emphasised the initiation of critical interventions instead of completeness of critical care. It might also reflect the triaging of critically ill women based on health workers’ rapid assessment of disease severity that is a common practice in busy hospitals. Nevertheless, our finding suggests that appropriate measures to incorporate other dimensions of quality, such as safety and equity, would need to complement timely use of effective interventions to improve maternal survival rate in these hospitals.

The analysis of avoidable factors contributing to substandard care clearly shows where concerted institutional efforts could improve maternal outcomes. Medical personnel‐related factors, unlike those related to women with SMO, can be addressed at institutional level to improve maternal survival within a short time frame. The documentation of personnel issues as the least frequent contributory factor to substandard care needs to be interpreted with caution, as it probably reflects under‐registration of lapses in care and further highlights the challenges of implementing critical incident audit in resource‐limited settings.^23^, ^24^ However, the fact that they were significantly more frequent among women who died than among those who survived suggests that more lives could be saved by improving how the medical teams deliver care. Likewise, as nearly one‐fifth of women with SMO had financial barriers to receiving care, it is clear that the Nigerian health system can significantly reduce inequity in care provision and improve maternal outcomes by revitalising the National Health Insurance Scheme.

### Conclusions and recommendations

Tertiary hospitals in Nigeria are underperforming in terms of their ability to promptly deliver quality care to ensure survival of critically ill women. To achieve a significant reduction in maternal mortality at this level, it is essential to address the noted deficiencies in care and at the same time strengthen the capacity of primary, secondary and private health facilities to prevent and manage obstetric emergencies. In settings where critically ill women present late to the hospital, maternal survival is not entirely dependent on the availability and application of life‐saving interventions. Rather, it requires the full complement of care that also includes safe, efficient and equitable use of such interventions. Health policy makers in Nigeria should urgently address the bottlenecks within the health system to reduce the delays in instituting comprehensive care. New national initiatives such as the Maternal Death Review project could leverage on the achievements of our network to establish a sustainable system of maternal death surveillance and response in Nigeria.^25^ Future research should assess completeness of care and other dimensions of quality to obtain a complete picture of where care improvement could save lives. Achieving global targets for maternal death post‐2015 by the international community would require prioritising Nigeria with a focus not only on increasing institutional birth but also on strategies to improve survival of women presenting for hospital care.

### Disclosure of interests

None declared. Completed disclosure of interests form available to view online as supporting information.

### Contribution to authorship

OTO conceived the study with inputs from OOA. The study implementation was a collaborative effort of a large number of academic staff, hospital personnel and researchers from 42 Nigerian tertiary hospitals—*Nigeria Near‐miss and Maternal Death Surveillance Network—*working under the auspices of the Nigerian Network for Reproductive Health Research and Training. OTO and OOA (co‐principal investigators) drafted the report with substantial contributions from BAE, CC, SE, APA, AA, OA, ASA, ABA, JII, JOI, OK, LO, OUJU, OAD, OT, JPV, and AMG (in order of authors). OTO, OOA, BAE, CC, SE, APA, HEO, AA, ANA, OA, ASA, COA, OA, LDA, ABA, AA, MB, AF, ADG, JII, JOI, OK, DN, LO, KT, JT, OUJU, ACU, OAD, OT, JPV, AMG (in order of authors) and individuals named under *Nigeria Near‐miss and Maternal Death Surveillance Network* reviewed the draft manuscript for intellectual content and approved the final manuscript for publication.

### Details of ethics approval

The WHO Research Ethics Review Committee (WHO ERC) reviewed and approved the study on 10 May 2011 (protocol ID: A65745, version 4). Ethics approval for continuation of the project was granted by the same committee on 3 May 2012. Ethics review authorities in the 42 participating hospitals reviewed and approved the study.

### Funding

The UNDP/UNFPA/UNICEF/WHO/World Bank Special Programme of Research, Development and Research Training in Human Reproduction (HRP), Department of Reproductive Health and Research, World Health Organization funded the study. The end‐of‐project meeting of all investigators was jointly supported by a grant from Merck, through its *Merck for Mothers* Programme, and the World Health Organization.

## Supporting information


**Figure S1.** Forest plot of intrahospital maternal mortality ratio (×10^3^) for all hospitals.

**Appendix S1.** Definition of terms.

**Appendix S2.** The WHO maternal near‐miss criteria.

**Table S1.** Demographic characteristics of women with life‐threatening maternal complications.

**Table S2.** Regional distribution of direct obstetric complications and corresponding cause‐specific case fatality rates.

**Table S3.** Maternal and perinatal outcomes and near‐miss indicators by regions.

**Table S4.** Intrahospital maternal mortality ratio by participating hospitals.

## Appendix 1

### Nigeria Near‐miss and Maternal Death Surveillance Network

OO Fakeye: Department of Obstetrics and Gynaecology, University of Ilorin Teaching Hospital, Ilorin, Kwara State, Nigeria.

D Abubakar: Department of Obstetrics and Gynaecology, Federal Medical Centre, Gusau, Sokoto State, Nigeria.

A Adeniyi: Department of Obstetrics and Gynaecology, Federal Medical Centre, Ido‐Ekiti, Ekiti State, Nigeria.

N Adewole: Department of Obstetrics and Gynaecology, University of Abuja Teaching Hospital, Gwagwalada, Federal Capital Territory, Nigeria.

A Adeyemi: Department of Obstetrics and Gynaecology, Ladoke Akintola University Teaching Hospital, Osogbo, Osun State, Nigeria.

A Agbata: Department of Obstetrics and Gynaecology, Federal University Teaching Hospital, Abakaliki, Ebonyi State, Nigeria.

B Ageda: Department of Obstetrics and Gynaecology, Federal Medical Centre, Makurdi, Benue State, Nigeria.

ES Aigere: Department of Obstetrics and Gynaecology, Federal Medical Centre, Katsina, Katsina State, Nigeria.

FC Anolue: Department of Obstetrics and Gynaecology, Imo State University Teaching Hospital, Orlu, Imo State, Nigeria.

A Bariweni: Department of Obstetrics and Gynaecology, Federal Medical Centre, Yenagoa, Bayelsa State, Nigeria.

F Ezugwu: Department of Obstetrics and Gynaecology, Enugu State University of Science and Technology Teaching Hospital, Enugu, Enugu State, Nigeria.

B Fawole: Department of Obstetrics and Gynaecology, University College Hospital, Ibadan, Oyo State, Nigeria.

K Hunyinbo: Department of Obstetrics and Gynaecology, Federal Medical Centre, Abeokuta, Ogun State, Nigeria.

C Kamanu: Department of Obstetrics and Gynaecology, Abia State University Teaching Hospital, Aba, Abia State, Nigeria.

G Maduakor: Department of Obstetrics and Gynaecology, Federal Medical Centre, Asaba, Delta State, Nigeria.

A Massa: Department of Obstetrics and Gynaecology, Federal Medical Centre, Gombe, Gombe State, Nigeria.

JY Moruppa: Department of Obstetrics and Gynaecology, Federal Medical Centre, Yola, Adamawa State, Nigeria.

EA Nzeribe: Department of Obstetrics and Gynaecology, Federal Medical Centre, Owerri, Imo State, Nigeria.

AN Ocheke: Department of Obstetrics and Gynaecology, Jos University Teaching Hospital, Jos, Plateau State, Nigeria.

AO Oguntayo: Department of Obstetrics and Gynaecology, Ahmadu Bello University Teaching Hospital, Zaria, Kaduna State, Nigeria.

T Onile: Department of Obstetrics and Gynaecology, Federal Medical Centre, Lokoja, Kogi State, Nigeria.

JO Sotunsa: Department of Obstetrics and Gynaecology, Olabisi Onabanjo University Teaching Hospital, Sagamu, Ogun State, Nigeria.

GO Ugwu: Department of Obstetrics and Gynaecology, University of Nigeria Teaching Hospital, Enugu, Enugu State, Nigeria.

I Wanonyi: Department of Obstetrics and Gynaecology, Federal Medical Centre, Jalingo, Taraba State, Nigeria.
