# Supplementary material for: When getting there is not enough: a nationwide cross‐sectional study of 998 maternal deaths and 1451 near‐misses in public tertiary hospitals in a low‐income country
Source: BJOG. 2015 May 14;123(6):928–38. doi: 10.1111/1471-0528.13450 (PMC5016783; doi:10.1111/1471-0528.13450)
Supplement: Supplementary file 1 — Figure S1. Forest plot of intrahospital maternal mortality ratio (×103) for all hospitals. [file BJO-123-928-s001.doc]

1-7: northcentral hospitals; 8-13: northeast hospitals; 14-20: northwest hospitals; 21-27: southeast hospitals; 28-33: southsouth hospitals; 34-42: southwest hospitals

**Figure S1: Forest plot of intrahospital maternal mortality ratio (x10^3^) for all hospitals (N=42)**
