# Supplementary material for: When getting there is not enough: a nationwide cross‐sectional study of 998 maternal deaths and 1451 near‐misses in public tertiary hospitals in a low‐income country
Source: BJOG. 2015 May 14;123(6):928–38. doi: 10.1111/1471-0528.13450 (PMC5016783; doi:10.1111/1471-0528.13450)
Supplement: Supplementary file 2 — Appendix S1. Definition of terms. [file BJO-123-928-s002.doc]

**Appendix S1: Definition of terms**

| - **Maternal near-miss (MNM)**: A woman who nearly died but survived a complication that occurred during pregnancy, childbirth or within 42 days of termination of pregnancy·^12^ - **Maternal death (MD)**: The death of a woman while pregnant or within 42 days of termination of pregnancy, irrespective of the duration and the site of the pregnancy, from any cause related to or aggravated by the pregnancy or its management, but not from accidental or incidental causes (according to the tenth International Classification of Diseases [ICD-10])·^13^ - **Underlying cause of death**: The disease or condition that initiated the morbid chain of events leading to death or the circumstances of the accident or violence that produced a fatal injury”·^13^ - **Live Birth (LB):** Birth of an offspring which breathes or shows evidence of life·^11^ - **Severe maternal outcome (SMO)**: The occurrence of life-threatening condition (i.e. organ dysfunction) resulting in a maternal death or maternal near-miss·^11^ - **Severe maternal outcome ratio (SMOR)**: The number of women with life-threatening conditions (i.e. maternal death and near-misses) per 1000 live births· This indicator gives an estimate of the amount of care and resources that would be needed in an area or facility [SMOR = (MNM + MD)/LB]·^11^ - **Maternal near-miss ratio (MNMR)**: the number of maternal near-miss cases per 1,000 live births (MNMR = MNM/LB)· Similarly to the SMOR, this indicator gives an estimation of the amount of care and resources that would be needed in an area or facility·^11^ - **Intra-hospital MMR**: The number of maternal death that occurred in the hospital per 100,000 live births·^11^ - **Mortality index (MI)**: The number of maternal deaths divided by the number of women with life-threatening conditions (i.e. maternal near-misses plus maternal deaths) expressed as a percentage [MI=MD/ (MNM+MD)]· The higher the index, the more the women with life-threatening conditions who die (indicative of low quality of comprehensive emergency care), whereas the lower the index the fewer women with life-threatening conditions who die (better quality of care)·^11^ - **Cause-specific mortality index**: the number of maternal deaths resulting from a particular life-threatening condition divided by the sum of maternal near-misses and maternal deaths occurring from such condition, expressed as a percentage·^11^ - **Cause-specific case fatality rate**: the proportion of women who died among all women that experienced a particular direct obstetric complication irrespective of severity·^14^ |
| --- |
