# Supplementary material for: When getting there is not enough: a nationwide cross‐sectional study of 998 maternal deaths and 1451 near‐misses in public tertiary hospitals in a low‐income country
Source: BJOG. 2015 May 14;123(6):928–38. doi: 10.1111/1471-0528.13450 (PMC5016783; doi:10.1111/1471-0528.13450)
Supplement: Supplementary file 3 — Appendix S2. The WHO maternal near‐miss criteria. [file BJO-123-928-s003.doc]

| **Appendix S2: The WHO maternal near miss criteria**: a woman presenting any of the following criteria life-threatening conditions and surviving a complication that occurred during pregnancy, childbirth or within 42 days of termination of pregnancy should be considered as a maternal near miss case^12^ | | | |
| --- | --- | --- | --- |
| **Dysfunctional system** | **Clinical criteria** | **Laboratory markers** | **Management based proxies** |
| **Cardiovascular** | Shock  Cardiac arrest | Severe hypoperfusion  (lactate>5 mmol/L or >45mg/dL)  Severe acidosis (pH<7·1) | Use of continuous vasoactive drugs  Cardio-pulmonary resuscitation |
| **Respiratory** | Acute cyanosis  Gasping  Severe tachypnea  (respiratory rate >40 bpm)  Severe bradypnea  (respiratory rate <6 bpm) | Severe hypoxemia  (Oxygen saturation < 90% for ≥ 60 minutes or PaO2/FiO2<200) | Intubation and ventilation not related to anaesthesia |
| **Renal** | Oliguria non responsive to fluids or diuretics | Severe acute azotemia  (creatinine ≥300μmol/l or ≥3·5 mg/dL) | Dialysis for acute renal failure |
| **Haematologic/ Coagulation** | Failure to form clots | Severe acute thrombocytopenia  (<50,000 platelets/ml) | Massive transfusion of blood / red cells (≥ 5 units) |
| **Hepatic** | Jaundice in the presence of preeclampsia | Severe acute hyperbilirubinemia  (bilirubin>100 μmol/l or  >6·0mg/dL) |  |
| **Neurologic** | Prolonged unconsciousness  (lasting >12h)  Stroke  Uncontrollable fit / status epilepticus  Global paralysis |  |  |
| **Alternative severity proxy** |  |  | Hysterectomy following infection or haemorrhage |

*a) Shock is a persistent severe hypotension, defined as a systolic blood pressure <90 mmHg for ≥60 minutes with a pulse rate at least 120 despite aggressive fluid replacement (>2L)*

*b) Cardiac arrest refers to the Loss of consciousness AND absence of pulse/heart beat*

*c) Gasping is a terminal respiratory pattern and the breath is convulsively and audibly caught·*

*d) Oliguria is defined as an urinary output <30ml/hr for 4 hours or <400ml/24hr*

*e) Clotting failure can be assessed by the bedside clotting test or absence of clotting from the IV site after 7-10 minutes*

*f) Loss of consciousness is a profound alteration of mental state that involves complete or near-complete lack of responsiveness to external stimuli· It is defined as a Coma Glasgow Scale <10 (moderate or severe coma)·*

*g) Stroke is a neurological deficit of cerebrovascular cause that persists beyond 24 hours or is interrupted by death within 24 hours*

*h) Pre-eclampsia is defined as the presence of hypertension associated with proteinuria· Hypertension is defined as a blood pressure of at least 140 mm Hg (systolic) or at least 90 mm Hg (diastolic) on at least two occasions and at least 4–6 h apart after the 20th week of gestation in women known to be normotensive beforehand· Proteinuria is defined as excretion of 300 mg or more of protein every 24 h· If 24-h urine samples are not available, proteinuria is defined as a protein concentration of 300 mg/L or more (≥1 + on dipstick) in at least two random urine samples taken at least 4–6 h apart*

*i) For instance, continuous use of any dose of dopamine, epinephrine or norepinephrine*
