# Supplementary material for: When getting there is not enough: a nationwide cross‐sectional study of 998 maternal deaths and 1451 near‐misses in public tertiary hospitals in a low‐income country
Source: BJOG. 2015 May 14;123(6):928–38. doi: 10.1111/1471-0528.13450 (PMC5016783; doi:10.1111/1471-0528.13450)
Supplement: Supplementary file 4 — Table S1. Demographic characteristics of women with life‐threatening maternal complications. [file BJO-123-928-s004.doc]

**Table S1: Demographic characteristics of women with life-threatening maternal complications**

**Characteristics Maternal near-miss Maternal death SMO**

N=1451 (%) N=998 (%) N=2449(%)

*Age (years)*

<20 150 (10·3) 88 (8·8) 238 (9·7)

20-35 1124 (77·5) 767 (76·9) 1891 (77·2)

>35 174 (12·0) 143 (14·3) 317 (12·9)

Data unavailable 3 (0·2) 0·0 (0·0) 0·0 (0·0)

*Marital status*

Married 1268 (87·4) 911 (91·3) 2179 (88·9)

Not married 175 (12·0) 79 (7·9) 254 (10·4)

Data unavailable 8 (0·6) 8 (0·8) 16 (0·7)

*Number of pregnancies*

1 331 (22·8) 249 (24·9) 580 (23·7)

2-5 761(52·4) 504 (50·5) 1265 (51·7)

>5 347 (23·9) 228 (22·9) 575 (23·5)

Data unavailable 12 (0·8) 17 (1·7) 29 (1·1)

Range 1-15 1-15 1-15

*Educational level*

No formal education 399 (27·5) 294 (29·4) 693 (28·3)

Primary school 306 (21·1) 200 (20·0) 506 (20·7)

Secondary school 423 (29·2) 214 (21·4) 637 (26·0)

Post-secondary school 212 (14·6) 109 (10·9) 321 (13·1)

Data unavailable 111 (7·6) 181 (18·0) 292 (11·9)

*Occupation*

Unemployed 747 (51·5) 473 (47·4) 1220 (49·8)

Unskilled 386 (26·6) 273 (27·4) 659 (26·9)

Semiskilled 181 (12·5) 114 (11·4) 295 (12·0)

Professional 93 (6·4) 42 (4·2) 135 (5·5)

Data unavailable 44 (3·0) 96 (9·6) 140 (5·7)

*Religion*

Islam 664 (45·8) 492 (49·3) 1156 (47·2)

Christianity 771(53·1) 487 (48·9) 1258 (51·4)

Traditional 6 (0·4) 4 (0·4) 10 (0·4)

Data unavailable 10 (0·7) 15 (1·5) 25 (1·0)

*Social class*

Low 1088 (75·0) 731 (73·3) 1819 (74·3)

Middle 270 (18·7) 164 (16·4) 434 (17·7)

High 37 (2·6) 17 (1·7) 54 (2·2)

Data unavailable 56 (3·9) 86 (8·6) 142 (5·8)

*Place of residence*

< 5 km to hospital 512 (35·3) 272 (27·3) 784 (32·0)

> 5 km to hospital 929 (64·1) 708 (70·9) 1637 (66·9)

Data unavailable 10 (0·7) 18 (1·8) 28 (1·1)

*Registration status*

Booked 313 (21·5) 222 (22·2) 535 (21·8)

Unbooked 1097 (75·6) 759 (76·1) 1856 (75·8)

Data unavailable 41 (2·8) 17 (1·7) 58 (2·4)

*Antenatal care status*

None 674 (46·4) 439 (43·9) 1113 (45·5)

Some form of ANC 439 (30·3) 332 (33·3) 771 (31·6)

ANC at study site 218 (15·0) 142 (14·2) 360 (14·7)

Data unavailable 120 (8·3) 85 (8·5) 205 (8·4)

*Referral status*

Not referred 725 (49·9) 433 (43·4) 1158 (47·3)

Referred before labour 258 (17·9) 202 (20·2) 460 (18·8)

Referred during labour 242 (16·7) 145 (14·5) 387 (15·8)

Referred postpartum 203 (14·0) 206 (20·6) 409 (16·7)

Data unavailable 23 (1·5) 12 (1·2) 35 (1·4)

*Time of admission*

8.00 a.m. to 6.00 p.m. 737 (50·8) 548(55·0) 1285 (52·5)

6.01 p.m. to 7.59 p.m. 699 (48·2) 442 (44·2) 1141 (46·6)

Data unavailable 15 (1·0) 8 (0·8) 23 (0·9)
