# Supplementary material for: When getting there is not enough: a nationwide cross‐sectional study of 998 maternal deaths and 1451 near‐misses in public tertiary hospitals in a low‐income country
Source: BJOG. 2015 May 14;123(6):928–38. doi: 10.1111/1471-0528.13450 (PMC5016783; doi:10.1111/1471-0528.13450)
Supplement: Supplementary file 5 — Table S2. Regional distribution of direct obstetric complications and corresponding cause‐specific case fatality rates. [file BJO-123-928-s005.doc]

**Table S2: Regional distribution of direct obstetric complications and corresponding cause-specific case fatality rates**

| **Primary complication** | **Northeast** | **Northwest** | **Northcentral** | **Southeast** | **Southwest** | **Southsouth** | **All complications** | **Maternal death** | **Cause-specific CFR (%)** |
| --- | --- | --- | --- | --- | --- | --- | --- | --- | --- |
| **Obstetric haemorrhage** |  | | | | | | | | |
| Placenta praevia | 251 | 328 | 208 | 203 | 322 | 343 | 1655 | 12 | **0·7** |
| Placental abruption | 368 | 330 | 124 | 135 | 200 | 167 | 1324 | 36 | **2·7** |
| Morbidly adherent placenta | 12 | 25 | 6 | 32 | 31 | 11 | 117 | 2 | **1·7** |
| Ruptured uterus | 114 | 203 | 73 | 126 | 81 | 116 | 713 | 87 | **12·2** |
| Postpartum haemorrhage | 391 | 409 | 320 | 281 | 470 | 216 | 2087 | 103 | **4·9** |
| Other obstetric haemorrhage | 37 | 10 | 67 | 42 | 28 | 50 | 234 | 4 | **1·7** |
| **Infection** |  |  |  |  |  |  |  |  |  |
| Puerperal sepsis | 123 | 130 | 93 | 63 | 141 | 75 | 625 | 67 | **10·7** |
| Chorioamnionitis | 17 | 29 | 28 | 45 | 47 | 75 | 241 | 17 | **7·1** |
| Pyelonephritis | 86 | 98 | 92 | 16 | 71 | 17 | 380 | 2 | **0·5** |
| Other systemic infection | 39 | 61 | 36 | 151 | 40 | 40 | 367 | 56 | **15·3** |
| **Abortive outcome** |  |  |  |  |  |  |  |  |  |
| Abortion-related haemorrhage | 993 | 744 | 552 | 702 | 384 | 554 | 3929 | 19 | **0·5** |
| Abortion-related infection | 94 | 63 | 63 | 59 | 135 | 93 | 507 | 46 | **9·1** |
| Ruptured ectopic | 143 | 206 | 233 | 156 | 383 | 222 | 1343 | 13 | **1·0** |
| **Hypertensive disorders** |  |  |  |  |  |  |  |  |  |
| Chronic hypertension | 222 | 133 | 190 | 103 | 203 | 175 | 1026 | 7 | **0·7** |
| Pre-eclampsia | 778 | 681 | 519 | 377 | 674 | 645 | 3674 | 69 | **1·9** |
| Eclampsia | 694 | 561 | 163 | 165 | 234 | 236 | 2053 | 213 | **10·4** |
| **Dystocia** |  |  |  |  |  |  |  |  |  |
| Prolonged labour | 167 | 207 | 127 | 307 | 216 | 176 | 1200 | 6 | **0·5** |
| Obstructed labour | 236 | 433 | 143 | 288 | 274 | 397 | 1771 | 19 | **1·1** |

CFR: Case fatality rate
