# Supplementary material for: When getting there is not enough: a nationwide cross‐sectional study of 998 maternal deaths and 1451 near‐misses in public tertiary hospitals in a low‐income country
Source: BJOG. 2015 May 14;123(6):928–38. doi: 10.1111/1471-0528.13450 (PMC5016783; doi:10.1111/1471-0528.13450)
Supplement: Supplementary file 6 — Table S3. Maternal and perinatal outcomes and near‐miss indicators by regions. [file BJO-123-928-s006.doc]

| **Maternal and perinatal outcomes** | **Northcentral**  **n=7** | **Northeast**  **n=6** | **Northwest**  **n=7** | **Southeast**  **n=7** | **Southsouth**  **n=6** | **Southwest**  **n=9** | **Total**  **n=42** |
| --- | --- | --- | --- | --- | --- | --- | --- |
| **Total births (n (%))** | 16861  (17·3) | 18928 (19·4) | 19372  (19·8) | 12732  (13·0) | 13676  (14·0) | 16065  (16·5) | 97634  (100·0) |
| **Live births (n (%))** | 16137  (17·6) | 17492  (19·1) | 18049  (19·7) | 11885  (13·0) | 12894  (14·1) | 15267  (16·6) | 91724  (100·0) |
| **Stillbirths (n (%))** | 724  (12·3) | 1436  (24·3) | 1323  (22·4) | 847  (14·3) | 782  (13·2) | 798  (13·5) | 5910  (100·0) |
| **Maternal near-miss (n (%))** | 219  (15·1) | 270  (18·6) | 242  (16·7) | 183  (12·6) | 217  (15·0) | 320  (22·1) | 1451  (100·0) |
| **Intra-hospital maternal death (n (%))** | 121  (12·1) | 169  (16·9) | 237  (23·7) | 94  (9·4) | 140  (14·0) | 237  (23·7) | 998  (100·0) |
| **Maternal death before arrival (n (%))** | 2  (1·5) | 9  (6·6) | 13  (9·6) | 43  (31·6) | 14  (10·3) | 55  (40·4) | 136  (100·0) |
| **SMO (n (%))** | 340  (13·9) | 439  (17·9) | 479  (19·6) | 277  (11·3) | 357  (14·6) | 557  (22·7) | 2449  (100·0) |
| **Indicators** |  |  |  |  |  |  |  |
| **MNM ratio (per 1000 live births)** | 13·6 | 15·4 | 13·4 | 15·4 | 16·8 | 21·0 | 15·8 |
| **SMO ratio (per 1000 live births)** | 21·1 | 25·1 | 26·5 | 23·3 | 27·7 | 36·5 | 26·7 |
| **Intra-hospital MMR (per 100000 live births)** | 750 | 966 | 1313 | 791 | 1086 | 1552 | 1088 |
| **Mortality Index (MD/ MNM+MD x 100%)** | 35·6 | 38·5 | 49·5 | 33·9 | 39·2 | 42·5 | 40·8 |

**Table S3: Maternal and perinatal outcomes and near-miss indicators by regions (‘n’ represents the number of hospitals)**

MD: maternal death; MNM: maternal near-miss; SMO: severe maternal outcome
