# Supplementary material for: When getting there is not enough: a nationwide cross‐sectional study of 998 maternal deaths and 1451 near‐misses in public tertiary hospitals in a low‐income country
Source: BJOG. 2015 May 14;123(6):928–38. doi: 10.1111/1471-0528.13450 (PMC5016783; doi:10.1111/1471-0528.13450)
Supplement: Supplementary file 7 — Table S4. Intrahospital maternal mortality ratio by participating hospitals. [file BJO-123-928-s007.doc]

**Table S4: Intrahospital maternal mortality ratio by participating hospitals**

| **Hospital** | **Live births** | **Maternal death** | **Intrahospital MMR (x10^3^)** | **Intrahospital MMR (x10^3^) (95% CI)** |
| --- | --- | --- | --- | --- |
| 1 | 2606 | 15 | 0·576 | 0·323 - 0·948 |
| 2 | 1715 | 3 | 0·175 | 0·0361 - 0·510 |
| 3 | 2520 | 24 | 0·952 | 0·611 - 1·414 |
| 4 | 1987 | 11 | 0·554 | 0·277 - 0·988 |
| 5 | 2929 | 31 | 1·058 | 0·720 - 1·499 |
| 6 | 2024 | 17 | 0·840 | 0·490 - 1·341 |
| 7 | 2356 | 20 | 0·849 | 0·519 - 1·308 |
| 8 | 3030 | 17 | 0·561 | 0·327 - 0·897 |
| 9 | 6158 | 64 | 1·039 | 0·801 - 1·325 |
| 10 | 2931 | 26 | 0·887 | 0·580 - 1·297 |
| 11 | 1560 | 41 | 2·628 | 1·892 -3·549 |
| 12 | 2095 | 13 | 0·621 | 0·331 - 1·059 |
| 13 | 1718 | 8 | 0·466 | 0·201 - 0·915 |
| 14 | 2198 | 20 | 0·910 | 0·557 - 1·402 |
| 15 | 5508 | 38 | 0·690 | 0·489 - 0·946 |
| 16 | 1616 | 41 | 2·537 | 1·827 - 3·426 |
| 17 | 3182 | 41 | 1·288 | 0·926 - 1·744 |
| 18 | 1097 | 25 | 2·279 | 1·480 - 3·346 |
| 19 | 2287 | 27 | 1·181 | 0·779 - 1·713 |
| 20 | 2161 | 45 | 2·082 | 1·523 - 2·777 |
| 21 | 1140 | 7 | 0·614 | 0·247 - 1·261 |
| 22 | 1171 | 11 | 0·939 | 0·470 - 1·675 |
| 23 | 2536 | 29 | 1·144 | 0·767 - 1·638 |
| 24 | 1062 | 8 | 0·753 | 0·326 - 1·479 |
| 25 | 1306 | 14 | 1·072 | 0·587 - 1·792 |
| 26 | 4268 | 13 | 0·305 | 0·162 - 0·520 |
| 27 | 402 | 12 | 2·985 | 1·552 - 5·156 |
| 28 | 2666 | 49 | 1·838 | 1·363 - 2·423 |
| 29 | 1672 | 7 | 0·419 | 0·168 -0·861 |
| 30 | 3032 | 18 | 0·594 | 0·352 - 0·937 |
| 31 | 1437 | 9 | 0·626 | 0·287 - 1·186 |
| 32 | 417 | 9 | 2·158 | 0·992 - 4·057 |
| 33 | 3670 | 48 | 1·308 | 0·966 - 1·730 |
| 34 | 520 | 10 | 1·923 | 0·926 - 3·508 |
| 35 | 3383 | 21 | 0·621 | 0·385 - 0·947 |
| 36 | 1893 | 54 | 2·853 | 2·150 - 3·706 |
| 37 | 1811 | 22 | 1·215 | 0·763 - 1·833 |
| 38 | 1788 | 29 | 1·622 | 1·089 - 2·321 |
| 39 | 1263 | 25 | 1·979 | 1·285 - 2·908 |
| 40 | 1178 | 20 | 1·698 | 1·040 - 2·610 |
| 41 | 866 | 20 | 2·309 | 1·416 -3·544 |
| 42 | 2565 | 36 | 1·404 | 0·985 - 1·938 |
| Total | 91724 | 998 | 1·139* | 0·957 - 1·338ǂ |
| **Intrahospital MMR from meta-analysis (I^2^=86·4%); ǂ95% confidence interval from meta-analysis* | | | | |
